# Does the unexpected death of the manikin in a simulation maintain the participants’ perceived self-efficacy? An observational prospective study with medical students

**DOI:** 10.1186/s12909-017-0944-x

**Published:** 2017-07-06

**Authors:** Anne Weiss, Morgan Jaffrelot, Jean-Claude Bartier, Thierry Pottecher, Isabelle Borraccia, Gilles Mahoudeau, Eric Noll, Véronique Brunstein, Chloé Delacour, Thierry Pelaccia

**Affiliations:** 1 Centre for Emergency Care Teaching (CESU 67), Strasbourg University Hospital, Strasbourg, France; 2 Prehospital Emergency Care Service (SAMU 67), Strasbourg University Hospital, Strasbourg, France; 3 College of the High Studies in Medicine (CHEM), Brest, France; 4 Emergency Departments Network in Alsace (RESURAL), Strasbourg, France; 5 Department of Simulation, Strasbourg Faculty of Medicine, Strasbourg, France; 6 Department of anesthesiology, Strasbourg University Hospital, Strasbourg, France; 7 Centre for Training and Research in Health Sciences Education (CFRPS), Strasbourg Faculty of Medicine, Strasbourg, France

**Keywords:** Emergency medicine, Simulation, Death, Perceived self-efficacy, Undergraduate medical students

## Abstract

**Background:**

The death of a simulated patient is controversial. Some educators feel that having a manikin die is prejudicial to learning; others feel it is a way of better preparing students for these situations. Perceived self-efficacy (PSE) reflects a person’s perception of their ability to carry out a task. A high PSE is necessary to manage a task efficiently. In this study, we measured the impact of the death of a simulated patient on medical students’ perceived self-efficacy concerning their ability to cope with a situation of cardiac arrest.

**Methods:**

We carried out a single-centre, observational, prospective study. In group 1 (*n* = 27), pre-graduate medical students were warned of the possible death of the manikin; group 2 students were not warned (*n* = 29). The students’ PSE was measured at the end of the simulated situation and after the debriefing.

**Results:**

The PSE of the two groups was similar before the debriefing (*p* = 0.41). It had significantly progressed at the end of the debriefing (*p* < 0,001). No significant difference was noted between the 2 groups (*p* = 0.382).

**Conclusions:**

The simulated death of the manikin did not have a negative impact on the students’ PSE, whether or not they had been warned of the possible occurrence of such an event. Our study helps defend the position which supports the inclusion of unexpected death of the manikin in a simulation setting.

**Electronic supplementary material:**

The online version of this article (doi:10.1186/s12909-017-0944-x) contains supplementary material, which is available to authorized users.

## Background

Simulation is an educational method which is used to expose learners to situations which are close to reality, in all its diversity, in an environment which is safe for both the patient and the health professionals [1, 2]. The educational benefit of the death of a manikin during a simulation is a controversial subject widely discussed in the literature [3]. A certain number of authors think that the manikin should never be made to die, whereas others recommend this practice. The former believe that using scenarios which lead to the death of the manikin increases student stress [4]. This is thought to have a negative impact on learning, notably by limiting the participants’ ability to transfer what they have learned in a simulated environment to the real-life environment [5, 6]. Moreover, this confrontation with death is thought to reduce some students’ desire to attend further simulation sessions [3]. The latter believe that confronting students with clinical situations in which the manikin shows an absence of vital signs makes them more efficient in handling such situations in their future professional practice, in the context of an emergency [7].

At the same time, work has revealed that medical and nursing students are not satisfied with the training provided in how to handle death and announcing bad news [8]. They do not feel adequately trained and prepared to face such treatment situations in real life [9–11]. A learner’s perception of their ability to carry out a task, termed “perceived self-efficacy” (PSE) by Bandura, is a decisive element in a person’s commitment to complete the task in question. On the one hand, it is a major determinant of the quality of learning. Many studies have shown that the higher the level of perceived self-efficacy, the more they choose activities that challenge them and give them the opportunity to develop their skills, and the higher the learning objectives they set [12]. They also better face the difficulties in order to persevere, better regulate their efforts, manage stress and anxiety, and attain a higher level of performance [12]. On the other hand, people tend to engage in tasks in which they feel competent and avoid those in which they don’t [13, 14]. A high PSE is therefore necessary in managing situations involving death so that students can face these situations and manage them effectively. According to Bandura, PSE is based on [15]:

- the subjects’ personal experiences
- observing colleagues onto whom the subject projects himself
- verbal persuasion; i.e. a judgment, whether positive or not, held by others concerning the tasks carried out by the person in question
- psychological and emotional states.

Situations experienced in simulation are likely to influence the PSE of students attending simulation sequences, with respect to the different determinants.

In terms of scenarios of critical clinical situations, the literature describes three types of situations which lead to an absence of vital signs on the simulator [16]:

- The expected death of the simulator, when trainers and learners know from the start that the manikin will lose its vital signs at a given moment. This type of scenario is often used to teach end of life situations with palliative care or oncology teams.
- Death resulting from an inappropriate action or failure to act by the student.
- Unexpected death, in which the trainer knows that the simulator will show an absence of vital signs. The learner will discover it during the development of the situation. Death then occurs as a result of an expected acute complication from an existing pathology and not as a consequence of an inappropriate action by the student.

The latter is more commonly encountered in emergency medicine, where its unexpected nature often leaves caregivers with the impression that they are helpless and inadequately trained to cope with this situation in both clinical practice, or even during a simulation session with a teacher and colleagues [17]. A certain number of studies have shown that simulation sessions can trigger intense emotions and stress in the participants [18]. It has been demonstrated that the two key phases of the simulation sequence in which work can be done to limit this stress are the prebriefing and the debriefing [19]. Leighton, in particular, felt that submitting students to a specific prebriefing on the possible death of the manikin can considerably reduce the development of negative emotions which lower a student’s perceived self-efficacy [16]. We hypothesized that medical students who are warned of the possibility of an evolution towards the death of the manikin during a simulated situation would improve their PSE compared with that of students who were not so warned.

## Methods

### Study design and population

After obtaining the approval of the Strasbourg faculty of medicine ethics committee, we carried out an observational, single-centre, prospective study. Medical students were subjected to a simulation session during which the manikin, simulating an acute pathology (cerebral hemorrhage), was programed to rapidly show an absence of vital signs. The absence of vital signs in the manikin corresponded to the presence of apnea combined with an absence of the carotid pulse. The students’ PSE relative to taking charge of situations of sudden death was assessed at the end of the simulated sequence and after the debriefing.

A total of 56 students consented to, and were included in the study. There were 34 women and 22 men. Eight simulation sessions were organized with groups of seven students per session. All subjects were undergraduate students, mostly in their final year of medical training. Students were randomized into two groups: the first group (Group 1) attended a specific prebriefing on the possible death of the manikin; the second group (Group 2) had a conventional prebriefing with no mention of the possible evolution of the simulator towards an absence of vital signs.

The patient was simulated by a high-fidelity manikin (Gaumard®) placed on a stretcher in an emergency room setting. The simulated session followed the usual three phases of this type of activity (Table 1), with an additional phase for group 1 learners of having a prebriefing describing the possible death of the manikin.

**Table 1:** Details of the themes covered by each phase of the simulation session and the groups concerned

Phases of the simulation session	Description of the phase	Groups
Briefing	Welcome, presentation of learners and teachers Presentation of the general training objectives Ensuring a context of well-being promoting learning and limiting student stress: *“Nothing that happens in a simulation session leaves the room; mistakes are allowed and they can be constructive; there is no judgment of value,* etc.*”* Invitation for participants to join a discussion process: *“By playing or actively observing the simulated session, you will note the actions carried out or not, and then try to analyze why, when and how they were performed”* Presentation of the instructor’s role Presentation of the Gaumard Hal manikin, the equipment and its limits	1 + 2
Specific prebriefing about the possible death of the manikin	Student information on the possible development of the absence of vital signs on the simulator, corresponding to patient death	1
Simulated sequence procedure		1 + 2
Distribution of the questionnaire assessing the students’ perceived self-efficacy in managing death situations		1 + 2
Debriefing	Respects the 3 conventional phases: - Reaction phase: word given to those involved to promote the emergence of emotions - Analysis phase: self-assessment, feedback between colleagues and teachers on the actions taken, so as to modify, improve or reinforce them, where necessary Opportunity to talk about sudden patient death and answer students’ questions - Summary: reformulation of key points of the patient’s treatment Message releasing students from feelings of guilt: *“The patient’s death was unavoidable. Don’t feel any kind of responsibility for this development and don’t feel guilty”.* Advice provided for managing this type of situation: *There is no recipe for handling patient death and announcing the news of this event to the family. Each case is unique, but if you need to know a few important points which could be helpful, it would be these:* - *Give yourself a moment to think and the decision to declare a patient dead* - *Rely on your team* - *Take your time in announcing it to the family* - *Let the family express themselves, let them ask questions and answer them, leave room for silence also* - *Be sympathetic* - *Share your experiences with your colleagues* - *Read documentation on this subject and ask for training*	1 + 2
Distribution of the questionnaire assessing the students’ perceived self-efficacy in managing death situations		1 + 2

The eight simulation sessions were presented by four experienced simulation instructors trained in debriefing. They were all given a detailed description of how the simulation session would proceed, in order to guarantee homogeneity from one group to the other. In each group, the prebriefing and debriefing were both led by the same instructor.

### Questionnaire design

We measured the students’ PSE relative to taking charge of situations of sudden death, using the Motivated Strategies for Learning Questionnaire (MSLQ) developed by Pintrich et al. [20], which has demonstrated high psychometric qualities (in terms of internal consistency, reliability and predictive validity) with college students [21]. It includes eight statements exploring perceived self-efficacy, which the student has to answer by stating their degree of agreement on an eight-item Likert scale. The higher the score, the higher the PSE. This initial questionnaire was translated into French, then, for each question, a proposal was made which was as close as possible to the first item and related to the task of managing a patient’s death (Table 2). We also asked the students if they had already received training in managing patient death and delivering bad news.

**Table 2:** Examples of items from the study questionnaire

Extracts from the study questionnaire	
I think I’m very efficient in handling the unexpected death of a patient	
I am certain to achieve the necessary skills to manage an unexpected death situation	

The questionnaire was given to each participant before the debriefing (pre-test) and at the end of the training session (post-test).

### Data analysis

We decided to strictly follow recommendations made by the authors of the MSLQ in terms of data analysis [20]. After entering the data into an Excel® table, they were imported into the R software® (version 3.1.3) to be analyzed. The test used to compare the two groups was a Chi2 analysis (with Yates continuity correction). When cohort numbers were too low, Fisher’s exact test was used. The groups were considered to be different if the *p*-value was less than 0.05. The Likert score was calculated by adding up the Likert items. The averages of the Likert scales for both groups were compared using Student’s t test. A difference of less than 5% (*p* < 0.05) was considered to be significant. The data are available as a supplemental file (Additional file 1).

## Results

The distribution of men and women was identical in both groups (*p* = 0.953). Students replied that they were confronted by the sudden death of a patient several times a year, with no difference between the two groups (*p* = 0.557). Group 1 had received more training than Group 2 in how to announce bad news (*p* = 0.016), whereas the level of training in managing a patient death was identical between the two groups (*p* = 0.232). The level of clinical experience between the two groups was similar (*p* = 0.557), with most of them being in their 6th year of medical training.

The PSE of the two groups was similar before the debriefing for all the items assessed (*p* = 0.41). The PSE also progressed significantly after the session (*p* < 0.001) (Table 3).

**Table 3:** Results of the PSE measurements in both groups

	PSE (Likert score) at the end of the simulated sequence	PSE (Likert score) at the end of the debriefing
Subgroup without specific prebriefing	average: 31.3 ± 8.1 median: 32	average: 37.8 ± 8.5 median: 36
Subgroup with specific prebriefing	average: 29.4 ± 8.7 median: 29	average: 35.5 ± 10.3 median: 36

Therefore, training appeared to have a positive effect on the evolution of the PSE. This progression occurred in the same proportions in both groups; there was therefore no significant difference between PSE levels, whether or not the group had been given the specific prebriefing (*p* = 0.382).

## Discussion

Our study revealed that undergraduate medical students near the end of their training, faced with a simulation of sudden patient death, improved their PSE relative to management of this type of situation. This study corroborates the position taken by Gordon et al. and by Gettman et al., according to whom training by simulation scenarios including the unexpected death of the manikin provides an opportunity for students to develop or improve their skills, with PSE constituting a necessary part of the efficient implementation of these skills [2, 22].

These results also reveal the potential benefits of exposing students to simulator death, as has already been reported by several other researchers [23–26]. To guarantee a positive learning situation, we followed the main recommendations found in the literature, including avoiding scenarios in which death results from an action or lack of action by the student, and using an experienced instructor (more than 2 years experience) [3, 16, 27]. We also paid particular attention to the care recommended during the prebriefing and the debriefing, including an appropriate amount of time for debriefing of not less than 30 min, to allow for adequate discussion about the thoughts which led to particular decisions or actions for the patient during the simulation. On the other hand, whereas Leighton [16] recommended paying particular attention to the prebriefing so as to limit the negative impact of such situations, we did not find any significant difference in perceived self-efficacy between the students in Group 1 (with prebriefing on the possible death of the manikin) and Group 2 (with no specific prebriefing). The impact of the prebriefing on PSE was therefore not established. On the other hand, we revealed the importance of debriefing in its ability to lead to an improvement in students’ PSE.

The fact that carrying out a prebriefing announcing the possible death of the manikin is not necessary is an interesting result. Not having to make such an announcement allows to maintain a form of authenticity to the simulation. The fact of drawing participants’ attention to this possible outcome may indeed lead them to increase their vigilance over the occurrence of this event, even though they would not have done so in a real situation. This could therefore compromise their clinical reasoning by pointing it to the signs to pay attention to during the sequence, even though recognizing the nature of the problem in a simulated or real clinical environment by picking up the relevant information is central to the medical expertise, particularly in the emergency medicine practice [28, 29]. This implies, if we want to train students to develop this skill, not to reveal the solution to the problem (e.g. by announcing the possible death of the manikin during the prebrefing) before the learners are confronted with it (e.g. when the manikin actually dies during the simulation).

Our study has some limitations. Apart from the single-centre aspect, the sample size was limited. In addition, the increase in PSE was not evaluated at any other point following the training in order to evaluate whether the learning from the session endured. Moreover, the questionnaire was translated from English into French, which may have impaired its psychometric qualities, although we have been particularly careful during this translation phase. This work should be continued in a multi-centre context, with a larger and more diversified population of students. Finally, we cannot exclude that filling out a questionnaire about PSE directly influences the respondents’ PSE and impacts on the students processing of the simulation experience.

## Conclusions

The originality of our study lies in the fact that we got interested in a particular aspect of the development and implementation of professional skills: perceived self-efficacy. We therefore wanted to provide original and unpublished light on whether or not exposing students to the death of a simulation manikin is useful or not.

Our work tends to corroborate the assertion made by some researchers of the benefits of exposing medical students to unexpected death when being trained in a simulated environment. Specifically, we showed that the absence of a prebriefing specifically surrounding simulated death has no negative effect on the PSE of undergraduate medical students relative to taking charge of situations of sudden death.

## Additional file

Additional file 1: Raw data, Data collected and analyzed in this study. (XLS 57 kb)

## Abbreviations

MSLQ: Motivated Strategies for Learning Questionnaire
PSE: Perceived self-efficacy
